# Is the association between precarious employment and mental health mediated by economic difficulties in males? Results from two Italian studies

**DOI:** 10.1186/s12889-019-7243-x

**Published:** 2019-07-03

**Authors:** Gianluigi Ferrante, Francesca Fasanelli, Antonella Gigantesco, Elisa Ferracin, Benedetta Contoli, Giuseppe Costa, Lidia Gargiulo, Michele Marra, Maria Masocco, Valentina Minardi, Cristiano Violani, Nicolás Zengarini, Angelo d’Errico, Fulvio Ricceri

**Affiliations:** 1 National Centre for Drug Research and Evaluation, National Institute of Health (ISS), Rome, Italy; 2 Epidemiologia&Precariato, Group for the study of precarious work of the Italian Association of Epidemiology (AIE), Rome, Italy; 3 Unit of Cancer Epidemiology, Città della Salute e della Scienza University-Hospital and University of Turin, Turin, Italy; 4 Centre of Behavioural Sciences and Mental Health, Italian National Institute of Health (ISS), Rome, Italy; 5 Unit of Epidemiology, Regional Health Service ASL TO3, Grugliasco (TO), Italy; 6 National Center for Disease Prevention and Health Promotion, National Institute of Health (ISS), Rome, Italy; 7 Department of Clinical and Biological Sciences, University of Turin , Grugliasco (TO), Italy; 8 National Institute of Statistics (ISTAT), Rome, Italy; 9 Department of Psychology, Faculty of Medicine and Psychology, University Sapienza, Rome, Italy

**Keywords:** Precarious work; mental health, Financial strain, Mediation analysis

## Abstract

**Background:**

Flexible employment is increasing across Europe and recent studies show an association with poor mental health. The goal of the current study is to examine this association in the Italian population to assess the possible mediating role of financial strain.

**Methods:**

Data were obtained by two Italian cross-sectional studies (PASSI and HIS) aimed at monitoring the general population health status, health behaviours and determinants. Mental health status was assessed using alternatively two validated questionnaires (the PHQ-2 and the MCS-12 score) and Poisson regression models were performed to assess if precarious work was associated with poor mental health. A formal mediation analysis was conducted to evaluate if the association between precarious work and mental health was mediated by financial strain.

**Results:**

The analyses were performed on 31,948 subjects in PASSI and on 21,894 subjects in HIS. A nearly two-fold risk of depression and poor mental health was found among precarious workers, compared to workers with a permanent contract, which was strongly mediated by financial strain.

**Conclusions:**

Even with the limitations of a cross-sectional design, this research supports that precarious employment contributes through financial strain to reduce the mental health related quality of life and to increase mental disorders such as symptoms of depression or dysthymia. This suggests that when stability in work cannot be guaranteed, it would be appropriate to intervene on the wages of precarious jobs and to provide social safety nets for ensuring adequate income.

**Electronic supplementary material:**

The online version of this article (10.1186/s12889-019-7243-x) contains supplementary material, which is available to authorized users.

## Background

Since the late 80’s in Europe precarious employment has increased steadily [1], following major legislative and economic changes in the organization of the labour market introduced in most European Union countries. In particular, in Italy, the «Treu law», enacted in 1997, promoted work flexibility allowing temporary contracts and redesigning the apprenticeship, but with the result of increasing the possibility of job insecurity [2]. Later in 2003, the «Biagi law» introduced additional forms of precarious employment, such as project-based and occasional contracts [2]. Due to these reforms, many permanent full-time jobs have been replaced by forms of precarious work with the consequence that in the last years among newly hired flexible contracts are almost the double of the permanent ones [3].

Early research on precarious work suggested that flexible employment could have positive effects for workers, allowing higher salaries, more prestigious positions and increased opportunities for professional experiences [4, 5]. However, this would apply only to high-skilled workers, whereas, in all other cases, it seems that flexible employment has rather negative consequences on both professional and private life as well as on health, due to structural uncertainty resulting from contractual uncertainty, income instability, and worse working conditions [6, 7]. Furthermore, more and more studies have documented, also in Italy [8], the health consequences of precarious employment, and most of these researches agree in attributing to job insecurity adverse effects on workers’ health, particularly mental health [6, 9, 10]. More generally, researchers agree that job insecurity has positive effects only if it is a voluntary choice and not a constraint [4].

It is also well-recognized that economic difficulties are an important determinant of health, especially mental health [11, 12]. During the 2008 global financial recession, that is known to have increased workers’ economic difficulties and reduced the possibility of finding a new job in case of dismissal, negative effects of the economic crisis on workers’ mental health and stress were observed [13]. Based on a review of 350 studies, a consensus paper of the European Psychiatric Association has recognized the deleterious consequences of the economic crises on mental health, particularly on psychological well-being, depression, anxiety, insomnia, alcohol abuse, and suicidal behavior. Unemployment, indebtedness, precarious working conditions, inequalities, lack of social connectedness, and housing instability emerge as main risk factors, especially for men of working age with a more disadvantaged socioeconomic position [14].

As mentioned above, many studies have explored the mental health effect of precarious work and economic strain separately, but the mediating role of economic strain still remains unclear.

Aims of this paper are to evaluate whether an association exists in the Italian population between precarious employment and mental health, as well as to assess the role of economic conditions as mediators of this relationship.

To test our hypotheses, we used the data from two different national sources: a) the National Health Interview Survey (HIS), conducted by the Italian National Institute of Statistics; b) the Italian behavioural risk factor surveillance system “Progressi delle Aziende Sanitarie per la Salute in Italia” (PASSI), conducted by the Italian National Institute of Health.

## Methods

### Study population

The HIS and PASSI are two cross-sectional studies conducted in Italy with the aim of monitoring the general population regarding health status, health behaviours and determinants, use of health services, and preventive measures for non-communicable diseases. Details of the two studies have been extensively described elsewhere [15, 16]. Briefly, the HIS is survey conducted every 5 years using a two-stage sampling design, whereby municipalities are sampled at first stage (with a proportional sampling probabilities given by size) and households at second stage. This survey is based on two different questionnaires: one asks about the family context and is administered face to face by an interviewer, whereas the other one is self-administered and concerns mainly health and lifestyles. The main topics included are lifestyles, perceived health status, work and economic situation of the individual, as well as the use of health services, including access to the emergency department, medical consultations, hospital admissions, and homecare assistance. The survey is carried out during four different quarters in order to take into account the seasonal effect.

PASSI is an ongoing surveillance system of behavioural risk factors, whose sample is extracted from the Italian adult population 18–69 years and the unit of sampling is represented by the Local Health Units (LHUs), which are the public structures responsible for preventive and curative services for residents in Italy. Trained LHU public health professionals administer telephone interviews through a standardized questionnaire and gather information on a wide variety of health-related behavioural and preventive topics, along with socio-demographic information. Interviews gathered during a calendar year, in order to take into account seasonal variations, are aggregated in an annual dataset.

For the current analysis we used the 2013 HIS survey and the 2014, 2015 and 2016 PASSI annual datasets combined. The population under study is that of workers aged 25–50 years holding any type employment, excluding those self-employed.

### Outcome definition

The mental health status in PASSI was assessed through the Patient Health Questionnaire 2 (PHQ-2), a 2-items depressive symptoms screening module [17]. This tool, validated through the Structured Clinical Interview for Diagnostic and Statistical Manual of Mental Disorders (fourth edition), an accepted gold standard, shows a sensitivity of 87% and a specificity of 78% for major depressive disorders [18]. The Italian version of PHQ-2 was validated by D’Argenio et al. [19]. In HIS, the mental health status was evaluated using the Mental health Component Summary (MCS-12) score of the Italian version of 12-item Short Form Health Survey (SF-12) questionnaire [20]. The SF-12 is a common, reliable and valid instrument for evaluating various aspects of health status experienced over the past 4 weeks, which includes both a physical and a mental component. MCS-12 overall scores, constructed from the individual items, are normalized to a range 0–100, with a population mean of 50 and a standard deviation of 10, using a proprietary weighting algorithm [20]. Since in the literature an acknowledged cut-off of the MCS-12 score for identifying probable diagnoses of common mental disorders is not available, we arbitrarily set the cut-off at the 5th percentile of the index distribution (MCS-12 = 32), which corresponds to the percentage of subjects with depressive symptoms in PASSI surveillance.

### Exposure definition

**Fig. 1:**
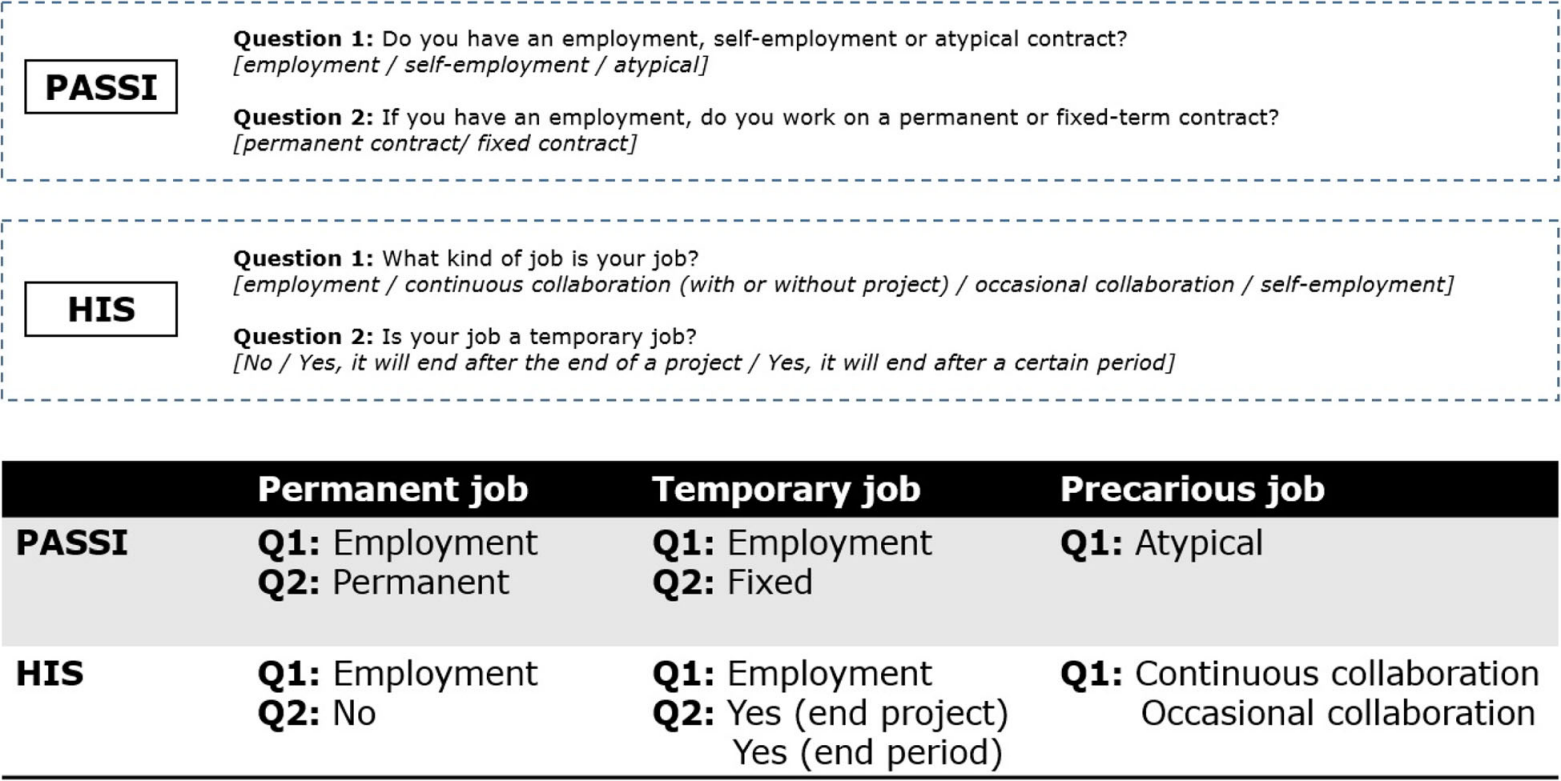
Definition of type of employment in each of the two studies

Using the questions on the type of employment in the two studies, three categories of employment were defined: i) subjects employed in a permanent job (Permanent); ii) subjects employed in a fixed-term job (Temporary); iii) subjects with a precarious job (Precarious). Both temporary and precarious jobs are based on fixed-term contracts, but the latter does not include, in addition, some workers’ rights, such as paid annual leave, paid sick leave, severance scheme, and complete pension contribution (Fig. 1).

### Covariates

Through the questionnaires we collected information on age, region of residence, marital status (married/partner cohabitant, single, widowed/divorced/separated-no partner cohabitant), living with children, occupational social class (large employers/salariat, intermediate, routine/manual, according to an aggregate version of the European Socio-economic Classification – ESeC [21]), economic sector (industry/construction, public employment, services, agriculture), education (primary/lower secondary school, higher secondary school, tertiary education), presence of at least one chronic condition. Moreover, the information on lack of financial resources (the mediator under study) was collected in PASSI through the question: «With the financial resources available, how do you get to the end of the month?». Those who reply: «with many difficulties» were considered people with economic difficulties. In HIS, it was defined on the basis of the question: «How easily can you to bear the essential costs?». Those who replied «hardly» were considered people with economic difficulties.

### Statistical analysis

All the analyses were performed separately for PASSI and HIS. Quantitative variables were described using mean and standard deviation (SD) and qualitative variables using absolute frequencies and percentages. Differences in covariates among types of employment (permanent, temporary, and precarious) were tested using one-way analysis of variance and chi-square test, for quantitative and qualitative variables, respectively. Prevalence ratios (PR) and corresponding 95% confidence intervals (95% CI) were calculated using Poisson regression with robust variance, weighting for a probability weight equal to the inverse of the sampling fraction, in order to obtain estimates generalizable to the Italian population [22]. Heterogeneity between the results of the two studies was evaluated using the I^2^ statistic, that describes the percentage of variation across studies due to heterogeneity, rather than to chance [23].

All tests were two-sided and the significance level was set to α = 0.05. All analyses were performed using STATA V13.

### Mediation analysis

The general idea of the mediation analysis is to decompose the total effect of an exposure (Total Causal Effect - TCE) on an outcome into a direct component that represents the direct effect of the exposure on the outcome (Pure Direct Effect - PDE) and in an indirect effect that is due to the effect of the exposure on a mediator and of the mediator on the outcome (Total Indirect Effect - TIE). In this study, TCE, PDE and TIE were estimated using the weighting approach [24] fitting a Poisson model with robust variance on the outcome (Additional file 1). The confounders considered in the mediation analysis were the same considered in the preliminary associations’ analysis.

## Results

The final analysis was performed on 31,948 subjects (51.8% males) in PASSI and on 21,894 subjects (53.9% males) in HIS. Differences among employment categories are presented in Table 1. As expected, in both studies, precarious workers were younger and showed higher prevalence of females, singles and subjects without children, compared to permanent workers. Interestingly, precarious workers also showed higher prevalence of subjects with higher occupational social class and with tertiary education. No differences among categories of employment were found regarding the prevalence of at least one chronic condition.

**Table 1:** Descriptive data of the two samples

Variable	PASSI Sample				HIS Sample			
	Permanent	Temporary	Precarious	*p*-value	Permanent	Temporary	Precarious	*p*-value
Age								
*Years, mean (SD)*	39.8 (6.9)	36.0 (7.5)	36.8 (7.6)	< 0.001	39.8 (6.8)	36.5 (7.3)	37.1 (7.3)	< 0.001
Gender								
*Male*	13,673 (53.6)	2317 (46.4)	551 (38.6)	< 0.001	9962 (55.2)	1606 (49.5)	235 (38.6)	< 0.001
*Female*	11,856 (46.4)	2676 (53.6)	875 (61.4)		8076 (44.8)	1642 (50,5)	373 (61.4)	
Region								
*North-West*	4588 (18.0)	684 (13.7)	214 (15.0)	< 0.001	4885 (27.1)	613 (18.9)	131 (21.5)	< 0.001
*Nort-East*	9360 (36.6)	1622 (32.5)	385 (27.0)		4506 (25.0)	685 (21.1)	99 (16.3)	
*Centre*	6557 (25.7)	1227 24.6)	369 (25.9)		3470 (19.2)	556 (17.1)	152 (25.0)	
*South*	3700 (14.5)	1014 (20.3)	359 (25.2)		3646 (20.2)	962 (29.6)	139 (22.9)	
*Islands*	1324 (5.2)	446 (8.9)	99 (6.9)		1531 (8.5)	432 (13.3)	87 (14.3)	
Marital status								
*Married/Partner cohabitant*	16,993 (66.6)	2653 (53.1)	771 (49.9)	< 0.001	10,034 (55.6)	1369 (42.1)	226 (37.2)	< 0.001
*Single*	6140 (24.0)	1951 (39.1)	572 (40.1)		6062 (33.6)	1523 (46.9)	307 (50.5)	
*Widowed/Divorced/Separated*	2396 (9.4)	389 (7.8)	143 (10.0)		1942 (10.8)	356 (11.0)	75 (12.3)	
Children								
*Yes*	10,840 (42.5)	1545 (30.9)	421 (29.5)	< 0.001	8140 (45.1)	1103 (34.0)	194 (31.9)	< 0.001
*No*	14,689 (57.5)	3448 (69.1)	1005(70.5)		9898 (54.9)	2145 (66.0)	414 (68.1)	
Occupational Social Class								
*Large employers and salariat*	2924 (12.7)	693 (15.0)	215 (16.4)	< 0.001	2119 (12.0)	513 (15.8)	140 (23.0)	< 0.001
*Intermediate*	13,068 (57.0)	2150 (46.5)	513 (39.1)		9396 (53.1)	1289 (39.7)	298 (49.0)	
*Routine and manual*	6953 (30.3)	1777 (38.5)	584 (44.5)		6175 (34.9)	1443 (44.5)	170 (28.0)	
Sector								
*Industries and constructions*	7692 (33.8)	1202 (25.9)	211 (16.0)	< 0.001	5527 (30.6)	721 (22.2)	83 (13.6)	< 0.001
*Services*	9173 (40.3)	2160 (46.6)	794 (60.1)		7383 (40.9)	1306 (40.2)	355 (58.4)	
*Public administration and health*	5545 (24.4)	985 (21.3)	229 (17.3)		4754 (26.4)	791 (24.4)	142 (23.4)	
*Agricolture*	328 (1.5)	288 (6.2)	87 (6.6)		374 (2.1)	430 (13.2)	28 (4.6)	
Education								
*None, primary or lower secondary*	5857 (23.0)	1364 (27.3)	412 (28.9)	< 0.001	6776 (37.6)	1402 (43.2)	196 (32.2)	< 0.001
*Higher secondary*	13,989 (54.8)	2282 (45.7)	629 (44.1)		7940 (44.0)	1084 (33.4)	212 (34.9)	
*Tertiary*	5678 (22.2)	1347 (27.0)	385 (27.0)		3322 (18.4)	762 (23.4)	200 (32.9)	
At least one chronic condition								
*Yes*	2718 (10.6)	507 (10.2)	148 (10.4)	0.57	2084 (11.5)	371 (11.4)	74 (12.2)	0.87
*No*	22,811 (89.4)	4486 (89.8)	1278(89.6)		15,954 (88.5)	2877 (88.6)	534 (87.8)	
Presence of economic difficulties								
*Yes*	1696 (6.7)	747 (15.0)	340 (23.9)	< 0.001	928 (5.1)	307 (9.5)	82 (13.5)	< 0.001
*No*	23,752 (93.3)	4239 (85.0)	1084(74.1)		17,110 (94.9)	2941 (90.5)	526 (86.5)	
Indication of mental illness								
*Yes*	945 (3.8)	190 (3.9)	70 (5.1)	0.05	801 (4.4)	155 (4.8)	38 (6.3)	0.09
*No*	23,982 (96.2)	4642 (96.1)	1305(94.9)		17,237 (95.6)	3093 (95.2)	570 (93.7)	

Table 2 shows the association between categories of employment and mental health status, separately by gender. Among men, in both surveys the likelihood of experiencing poor mental health was almost doubled in precarious compared to permanent workers, when the model was set with minimal adjustment. In the fully adjusted model, this association was slightly weaker but still significant in PASSI study (PR 1.90, 95% CI 1.08–3.34), while it lost statistical significance in HIS study (PR 1.69, 95% CI 0.91–3.16). However, no heterogeneity was found between the two surveys. In contrast, no difference in mental health was found for temporary male workers and for both temporary and precarious female workers, compared to permanent workers. Results did not change considering the subsample of single females, or that of single females with children (data not shown). Due to this lack of association in females, the mediation analysis was performed only in males.

**Table 2:** Association between type of employment and mental health

	PASSI Sample		HIS Sample		I^2^ for heterogeneity
	PR	95% CI	PR	95% CI	
MALES					
Model 1^a^					
*Permanent*	Ref		Ref		
*Temporary*	1.35	0.93–1.96	1.03	0.73–1.45	0.0%
*Precarious*	1.96	1.14–3.35	1.85	0.99–3.47	0.0%
Model 2^b^					
Permanent	Ref		Ref		
Temporary	1.41	0.95–2.08	1.02	0.72–1.45	22.6%
Precarious	1.90	1.08–3.34	1.69	0.91–3.16	0.0%
FEMALES					
Model 1^a^					
*Permanent*	Ref		Ref		
*Temporary*	0.98	0.75–1.28	1.23	0.97–1.55	35.7%
*Precarious*	1.10	0.76–1.62	1.19	0.77–1.83	0.0%
Model 2^					
Permanent	Ref		Ref		
Temporary	0.87	0.66–1.13	1.23	0.96–1.58	69.6%
Precarious	0.94	0.63–1.41	1.22	0.79–1.88	0.0%

^a^Robust Poisson regression model adjusted for age and region

^b^Robust Poisson regression model adjusted for age, region, marital status, living with children, occupational social class, economic sector, education, presence of at least a chronic condition

**Fig. 2:**
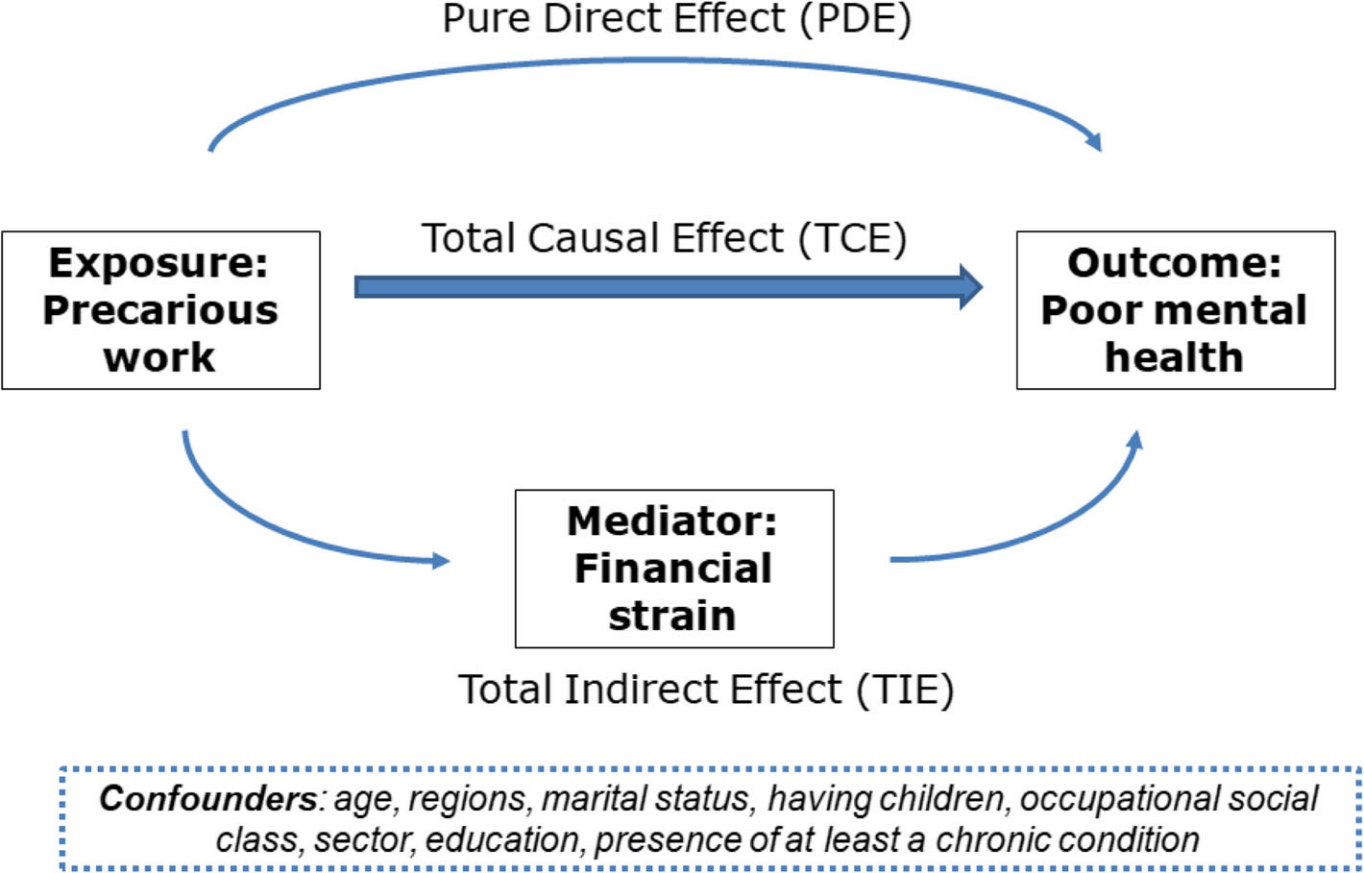
The mediation model proposed

The proposed mediation model is shown in Fig. 2. Its aim is to decompose the TCE between categories of employment and mental health into the PDE and the TIE through the mediation of the variable “lack of financial resources”. A strong TIE of financial strain was observed in PASSI (PR 1.49, 95% CI 1.31–1.72) and a weaker, although statistically significant, in HIS (PR 1.12, 95% CI 1.01–1.28), whereas PDE for precarious work was not anymore significant taking into account the presence of the mediator. Also, a statistically significant TIE was found for temporary work, limited to the PASSI sample (Table 3).

**Table 3:** Results of the mediation analysis. Analyses adjusted for age, region, marital status, living with children, occupational social class, economic sector, education, presence of at least one chronic condition

	PASSI Sample		
	Permanent	Temporary	Precarious
Pure direct effect (PDE)	Ref	1.15 (0.76–1.48)	1.28 (0.74–2.08)
Total indirect effect (TIE)	Ref	1.20 (1.13–1.29)	1.49 (1.31–1.72)
Total causal effect (TCE)	Ref	1.38 (0.95–1.82)	1.92 (1.09–2.93)
	HIS Sample		
Pure direct effect (PDE)	Ref	0.97 (0.74.1.25)	1.39 (0.87–1.94)
Total indirect effect (TIE)	Ref	1.02 (0.98–1.06)	1.12 (1.01–1.28)
Total causal effect (TCE)	Ref	0.99 (0.75–1.34)	1.57 (0.94–2.17)

## Discussion

The results of our analyses showed that Italian male workers with precarious employment are more likely to have mental health problems than those with permanent employment, while this phenomenon is not observed among temporary workers. Furthermore, we found that precarious job is not directly associated with poor mental health, but rather it is related with economic problems, possibly caused by job instability. The results described above only apply to males and were not observed in females.

A large body of evidence published in the literature in recent years demonstrates a link between job insecurity and mental health, which has been observed also in longitudinal studies [6, 8, 25, 26]. Strong associations have been found between job insecurity and minor psychiatric morbidity [27], mental distress [28] and poor mental health [29, 30], while other studies have shown the role of job instability in anxiety disorders [31] and in depression [26]. Many of these studies did not explore the results separately by gender, but where the outcomes are reported distinctly for males and females, some of them do not find gender differences [27, 32]. This could be due to the fact that these studies were conducted in Northern Europe countries, where the contribution of males and females to the family economic income is much more balanced than in Italy [33], where the male breadwinner model probably still stands as the most diffuse in society. In such a cultural context, it is plausible to assume that men perceive more strongly the problem of job insecurity or unemployment compared to women. In support of this hypothesis, a study conducted in another Southern Europe country (Spain) showed no effect of job insecurity on mental health in women, while an association was found in men [30]. Moreover, even restricting the analysis to single women, or to single women with children, where the breadwinner model might be expected, we did not find any association between type of employment and poor mental health. Interaction between biological and social factors could be an alternative explanation for the observed gender differences in Italy, but the literature evidence on this last hypothesis is divergent and non-final. It would be appropriate to further investigate the phenomenon through more suitable tools than those available to us.

Considering the role of financial strain in the relationship between precarious work and mental health, we found that the latter is not directly associated with poor mental health but there is a mediating factor, namely financial strain, which explains this relationship in men.

Actually, the association between scarcity of economic resources and poor mental health or poor perceived health status is not new information. Many publications have been produced on the subject, especially during the years of the global economic crisis: two systematic review on mental health outcomes show that economic recession is possibly associated with negative effects on psychological wellbeing, common mental disorders, substance disorders and suicidal behaviour [13, 34]. Bacci et al. demonstrated that indicators of economic deprivation are strong predictors of negative self-perceived health status [35].

With this study we add a further piece of knowledge showing that work precariousness is not directly associated with poor mental health, but it seems to act through financial strain.

The same mediation analysis conducted in the two surveys gave consistent results, but in PASSI the mediated effect of the economic difficulties was much stronger than in HIS. This difference could be due to the different mental health detection tools used in the two surveys. In fact, PASSI uses the PHQ-2, which is a depression-screening tool investigating symptoms of depressed mood and anhedonia. It has good reliability, validity, and sensitivity [18] and it was found to effectively rule out depression [36]. The MCS-12 was designed for use in the general population as a multi-factorial measure of health-related quality of life experienced over the past 4 weeks. The MCS-12 does not specifically assess depressive symptoms and, although related to depressive symptoms or diagnosed depression, is a measure less accurate of depression compared to PHQ-2 (e.g.: anhedonia is not investigated by the SF-12). The MCS-12 score does not target a specific psychiatric condition, but it is a more general indicator of mental well-being.

However, having obtained similar results in two large surveys regarding the effect of financial strain mediation in the association between precarious work and poor mental health can be considered a strong point in support of our conclusions. Furthermore, as far as we know, this is the first time that the role of financial strain in this association has been studied through a formal mediation analysis. This technique allows not only to evaluate the risk attenuation produced by the inclusion of the candidate mediator in a regression model, but also to quantify the component of the association mediated by the mediator.

The main limitation of our research is that data used for the analysis came from cross-sectional studies, whose design does not allow to infer a causal relationship between job insecurity and mental health. It is possible, in fact, that part of the association identified is due to reverse causality, as subjects with poor mental health could have been more likely to obtain a precarious work. However, in this case, the gender differences in this association would not be easily explainable. In addition, the cross-sectional design does not guarantee the assumption of temporality necessary to perform an unbiased mediation analysis. However, the relationship between exposure and outcome, exposure and mediator, and mediator and outcome have been already observed in longitudinal studies [37–39], making plausible our assumptions. Another limitation is that our results are based on self-reported data. In fact, the information collected by both PASSI and HIS is not based on objective measurements, but on answers provided by respondents. Therefore, the information collected can be influenced by various biases, especially when the topic of investigation is mental health, because of the social stigma surrounding this subject, leading to an underestimation of the prevalence. However, this bias may probably be a non-differential one and could lead at most to an attenuation of the strength of the association.

## Conclusions

Economic uncertainty is a factor strongly associated with poor mental health. Precarious employment with fixed-term contracts and low wages contributes to the economic strain, which in turn has consequences on mental health related quality of life and on the risk of symptoms of depression or dysthymia. These results have also some implications for employment and labour market policies: when job stability cannot be guaranteed for economic, political and market reasons, active labour market policies should be strengthened (by increasing their effectiveness, targeting, coverage) and their interaction with passive measures encouraged. Concerning passive policies, beside more traditional policies such as economic support for unemployment (subsides) and minimum salary set for precarious workers (currently under discussion in Italy), additional services such as access to mental health supporting cares should be guaranteed.

## Additional file

Additional file 1: In this file, the theoretical explanation of mediation analysis is presented. (DOCX 14 kb)

## Data Availability

The datasets analyzed in the current study are not publicly available because they belong to institutional entities such as the Italian Regions and the National Institute of Statistics, but are obtainable from the corresponding author on reasonable request. The authors of this work have access to the datasets since they are part of the network of researchers authorized to manage and analyze them.

## Abbreviations

CI: Confidence interval
ESeC: European Socio-economic Classification
HIS: Health Interview Survey
LHU: Local Health Unit
MCS-12: 12-item Mental health Component Summary
PASSI: *Progressi delle Aziende Sanitarie per la Salute in Italia*
PDE: Pure Direct Effect
PHQ-2: 2-item patient health questionnaire
PR: Prevalence
SD: Standard deviation
SF-12: 12-item Short Form Health Survey
TCE: Total Causal Effect
TIE: Total Indirect Effect
