# Supplementary material for: Is the association between precarious employment and mental health mediated by economic difficulties in males? Results from two Italian studies
Source: BMC Public Health. 2019 Jul 3;19:869. doi: 10.1186/s12889-019-7243-x (PMC6609380; doi:10.1186/s12889-019-7243-x)
Supplement: Supplementary file 1 — In this file, the theoretical explanation of mediation analysis is presented. (DOCX 14 kb) [file 12889_2019_7243_MOESM1_ESM.docx]

Supplementary material

Mediation analysis

Let’s suppose to have an exposure X that acts on an outcome Y, possibly through a mediator M and let’s suppose for simplicity that X, M, and Y are binary variables. Each subject has two counterfactual events: Y1, when the subject has been exposed to X and Y0, when the subject has not been exposed to X and likewise two counterfactual mediators: M1 and M0. Then, Yxm is the counterfactual event for the scenario X=x and M=m. The Total Causal Effect (TCE) is the difference E(Y1M1-Y0M0) and expresses how much the outcome would change in mean if the exposure were changed from level 0 to level 1 uniformly in the population. The Pure Direct Effect (PDE) is the contrast between the counterfactual outcome if the individual were exposed at X and the counterfactual outcome if the same individual were not exposed at X, with the mediator assuming whatever value it would have taken at the reference value of the not exposure. The Total Indirect Effect (TIE) is the contrast, having set the exposure as present, between the counterfactual outcome if the mediator assumed whatever value it would have taken at a value in the presence of exposure and the counterfactual outcome if the mediator assumed whatever value it would have taken in the absence of exposure. TCE, PDE and TIE were estimated using the weighting approach [22] fitting a Poisson model with robust variance on the outcome. The confounders considered in the mediation analysis were the same considered in the preliminary associations’ analysis.

Bias-corrected 95% confidence interval have been computed for the estimated, as they are suggested as best confidence intervals in boostrap procedure [23].
